# An uncommon variant of cyanotic congenital heart disease in a young adult female: a rare case of right pulmonary artery to left atrial fistula (PALAF)

**DOI:** 10.1186/s13104-015-1593-y

**Published:** 2015-10-23

**Authors:** Ram Sundar Twayana, Sanjaya Humagain, Rajendra Koju, Kriti Subas Joshi, Robin Man Karmarcharya, Sajana KC, Navaraj Poudel

**Affiliations:** Department of Cardiology, Dhulikhel Hospital, Kathmandu University Hospital, Dhulikhel, Nepal; Department of Radiology, Dhulikhel Hospital, Kathmandu University Hospital, Dhulikhel, Nepal; Department of Cardiovascular Surgery, Dhulikhel Hospital, Kathmandu University Hospital, Dhulikhel, Nepal; Department of Internal Medicine, Dhulikhel Hospital, Kathmandu University Hospital, Dhulikhel, Nepal; Department of Cardiology, Manipal College of Medical Sciences, Pokhara, Nepal

**Keywords:** Congenital, Left atrium, Pulmonary artery

## Abstract

**Background:**

Cyanotic congenital heart disease is not a rare entity, but fistula between the right pulmonary artery and the left atrium is an uncommon vascular anomaly. Although it is a real challenge to diagnose the case, detailed clinical evaluation and selective investigations are keys for diagnosis, and surgical intervention is still considered the best treatment option.

**Case presentation:**

A 19 years old girl from the remote village of Nepal presented with the history of exercise intolerance associated with cyanosis and clubbing of the extremities. We diagnosed her as a case of right pulmonary artery to left atrial fistula, a rare variant of pulmonary arteriovenous malformation. She underwent successful surgical correction of the anomaly under cardiopulmonary bypass surgery.

**Conclusion:**

Direct communication between the right pulmonary artery and the left atrium is a rare cyanotic congenital heart disease, which is diagnosed late and often associated with the atrial septal defect. The best treatment available is surgical correction.

## Background

Cyanotic congenital heart disease is not a rare entity but fistula between the right pulmonary artery and left atrium is an uncommon vascular anomaly. A direct shunt between the right pulmonary artery and the left atrium in the presence of intact atrial septum is a rare entity and diagnosis is yet another challenge. In the presenting case, there is an additional angiolipoma in the right posterior chest wall, which we considered as an incidental finding.

## Case presentation

A 19 years old girl from the remote village of Nepal with body mass index (BMI) of 19.5 presented to Kathmandu University Hospital, Dhulikhel Hospital complaining of exercise intolerance for several months and bluish discoloration of extremities and lips. She also noticed bilateral bulbous enlargements of the distal end of the fingers and toes. However, there were no significant clinical events during birth, post-natal and childhood periods.

**Fig. 1:**
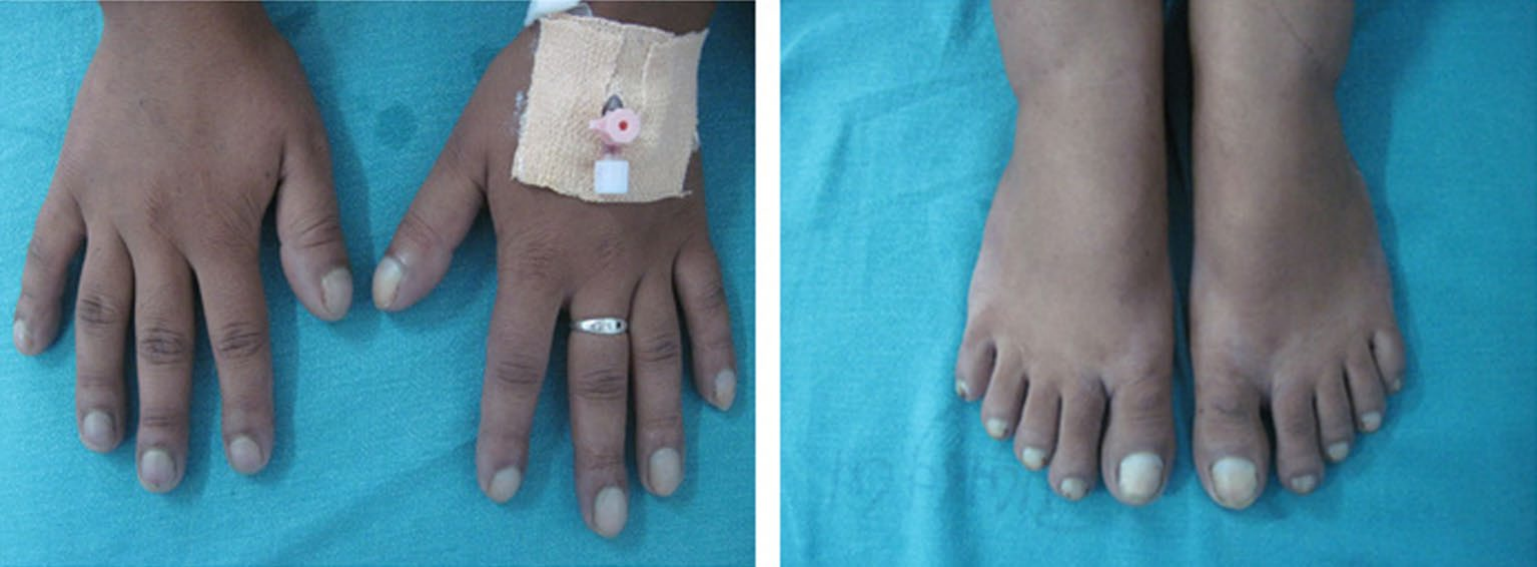
Showing bilateral grade III clubbing with cyanosis of the extremities

Clinical examinations revealed central and peripheral cyanosis with grade III clubbing of all extremities (Fig. 1) and plethoric lower conjunctiva. However, other systemic examinations showed no abnormal findings.

**Fig. 2:**
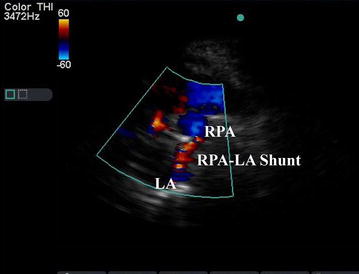
Transthoracic echocardiography showing shunt. *RPA* right pulmonary artery, *LA* left atrium

**Fig. 3:**
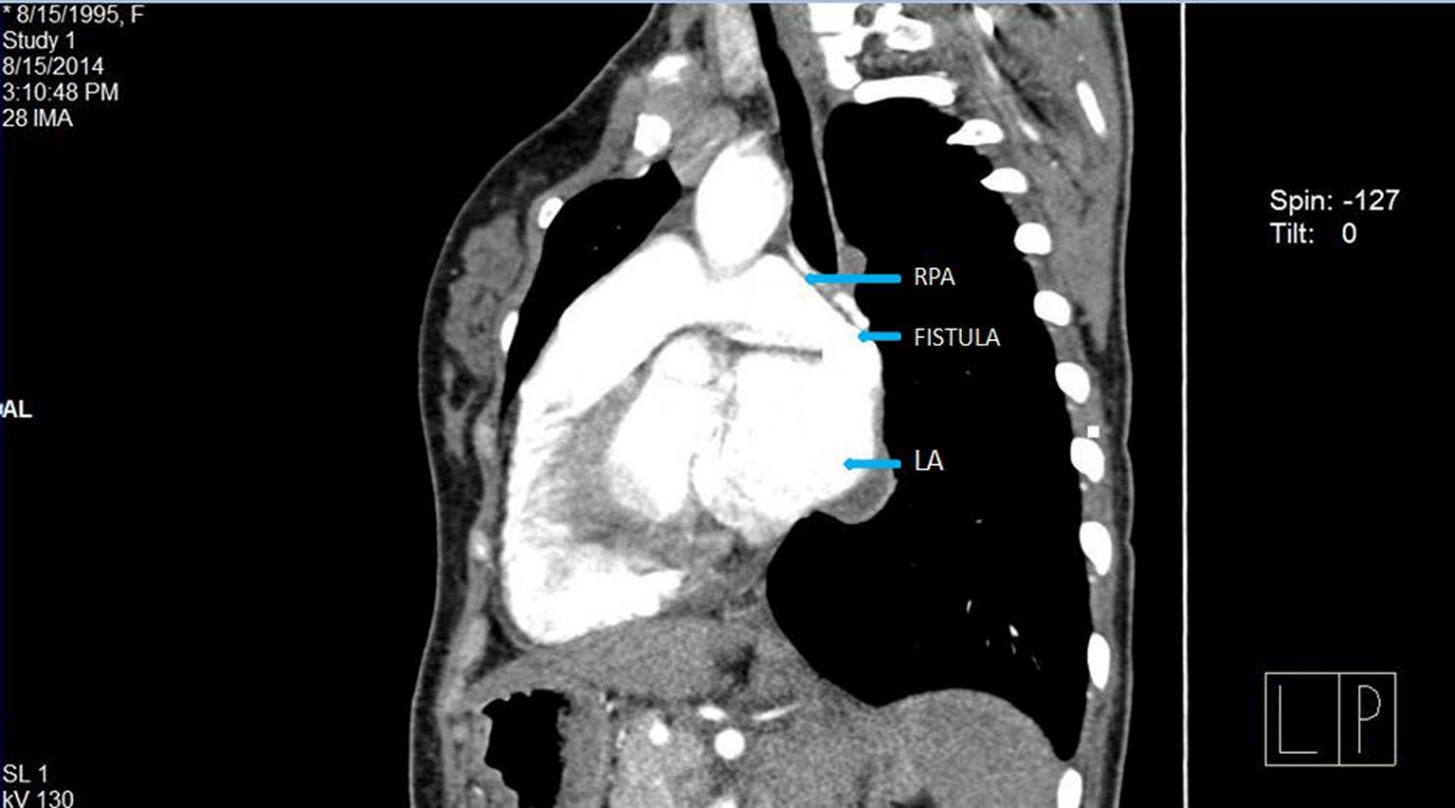
Contrast enhanced CT chest showing shunt between right pulmonary to left atrium (oblique view)

**Fig. 4:**
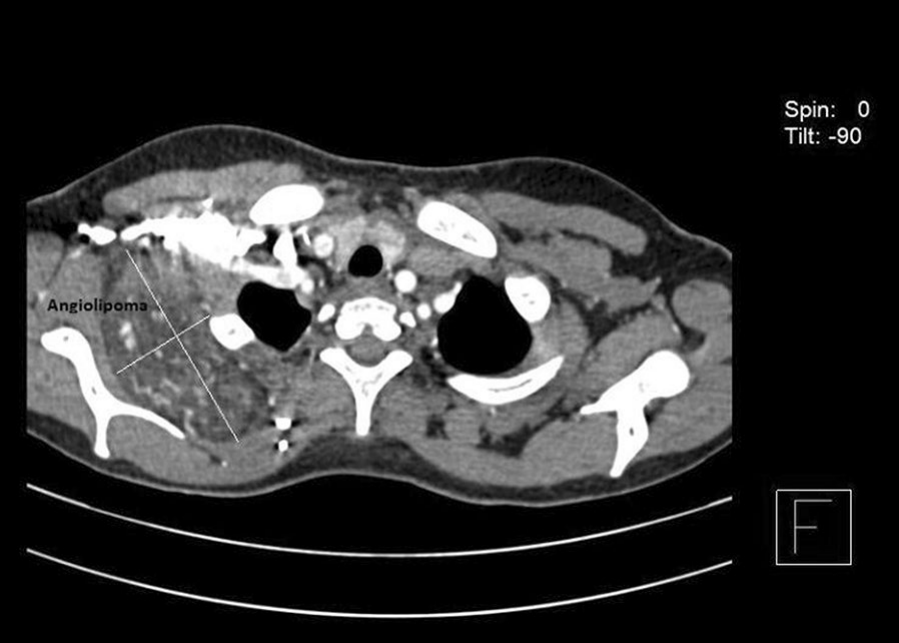
Angiolipoma between scapula and chest wall (right side)

**Fig. 5:**
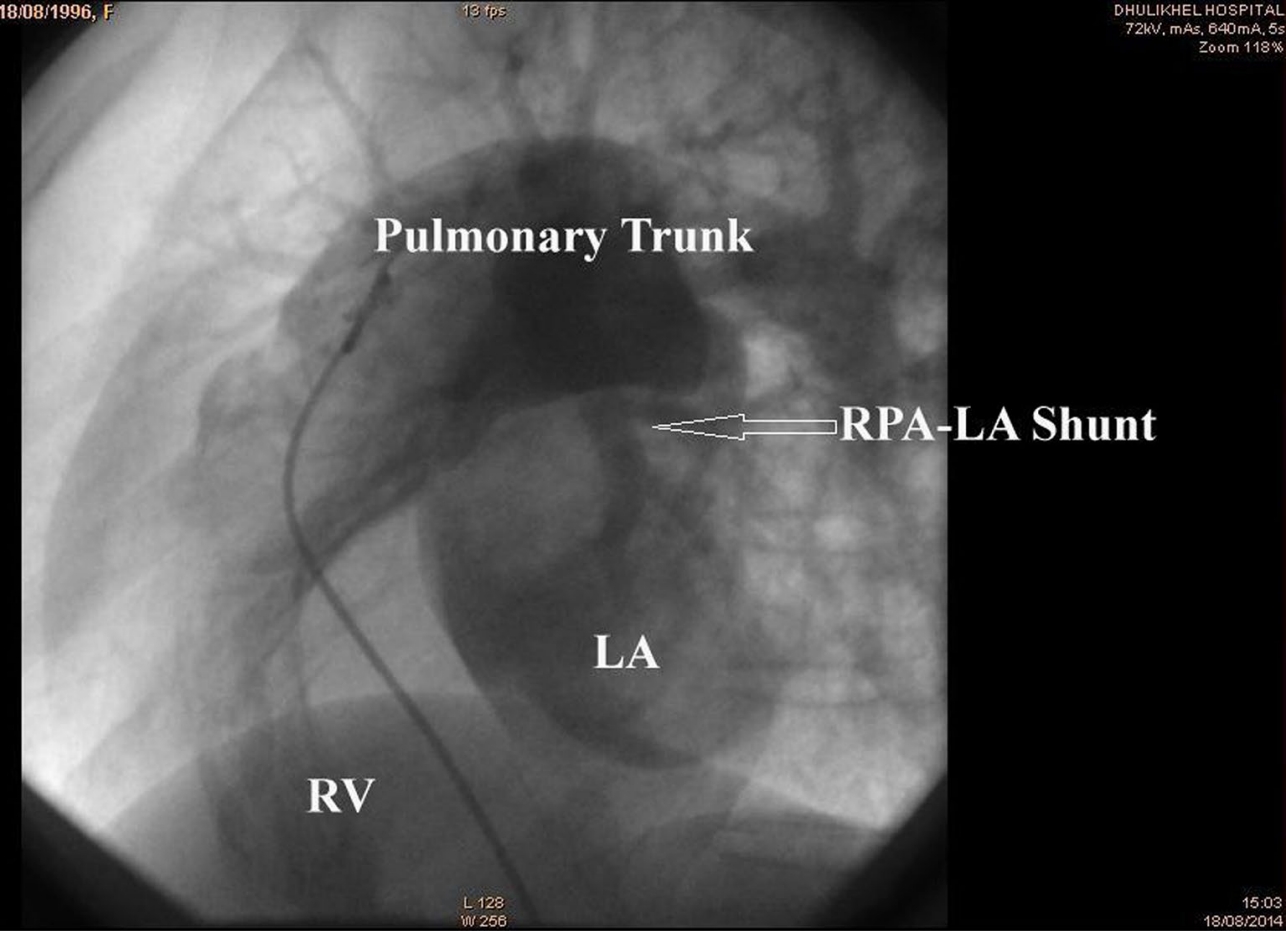
Pulmonary arteriogram showing shunt

Investigations showed oxygen saturation at 60 % at room air, which was not corrected by oxygen support. The arterial blood gas analysis report showed the partial pressure of oxygen (PaO_2_) at 44 mmHg, venous haemoglobin and haematocrit were 18.6 gm% and 62.2 %, respectively. Transthoracic echocardiography depicted mosaic flow (Fig. 2) from the right pulmonary artery to the left atrium (RPA-LA), and grade I pulmonary regurgitation. Chest radiograph and pulmonary function test were normal. Computed tomography (CT) of the chest showed enlarged left atrium and the direct communication from the posterior part of the right pulmonary artery into the left atrium, approximately 25 mm in size (Fig. 3). There was an additional angiolipoma (77–55 mm) on the right posterior chest wall between the scapula and the chest wall, which we consider an incidental finding (Fig. 4). We performed pulmonary arteriogram, which demonstrated the flow of the contrast from the abnormal branch of the right pulmonary artery to the left atrium (Fig. 5). The PaO_2_ in the pulmonary trunk and the right pulmonary artery, close to the shunt were 32 and 34 mmHg, respectively.

After confirming the diagnosis of the right pulmonary artery to the left atrial fistula, she underwent open heart surgery in National Heart Centre. Intra-operative finding showed a direct connection between the right pulmonary artery to the left atrium approximately 1.5–1.3 cm in size, which was closed using polytetrafluoroethylene patch. Immediately after closer of the shunt, her condition improved with adequate oxygen saturation at 95 % at room air. Her post-operative course was uneventful and discharged. She is now on regular follow up.

## Discussion

One of the rare causes of cyanotic congenital heart disease is a direct communication between RPA-LA, which was first described by Friedlich et al. [1]. This acts as a right to left shunt resulting in cyanosis, dyspnoea on exertion and polycythemia. In addition, the shunt bypasses the capillaries beds and permits emboli or bacteria to enter directly into the systemic circulation causing a stroke or cerebral abscess [2]. In most reported cases, the anomaly arises from the posterior part of the right pulmonary artery connecting the left atrium, between the right and the left pulmonary veins, which is similar in this case. Other commonly associated cardiac anomaly is atrial septal defect thus, causing a chance of missed diagnosis [3]. However, anomalies of the right lung such as an absence of middle or lower lobe, right lung sequestration, and diverticulum of the right main bronchus have also been reported [4]. Symptoms usually present in late adolescent unless the shunt is large with severe heart failure. The oldest age reported with this anomaly was 45 years [5].

Diagnosis is a real challenge for such anomaly. Echocardiography and contrast-enhanced CT are major investigations for conclusive diagnosis. In the presented case, we found unusual enlargement of the left atrium and mosaic flow from RPA-LA. Contrast-enhanced CT chest was done which revealed the exact anatomical defect of a direct communication between the posterior part of the right pulmonary artery and the left atrium. There was an incidental association of angiolipoma in the right thoracic cage. Cardiac catheterization and selective angiography along with measurements of partial pressure of oxygen from the pulmonary artery, pulmonary veins and the left ventricle assisted in confirming the diagnosis.

Although transcatheter embolization would be accepted better treatment for patient with arteriovenous malformation, the significant number of complications outweigh the open heart surgery thus, a majority of PALAF cases have undergone surgical repair [6]. Elective surgery is the best treatment to prevent chronic arterial hypoxemia, systemic thromboembolism and pulmonary hypertension. Nevertheless, the surgical procedure has a considerable mortality risk (22 %) and should be approached with due caution.

## Conclusions

In summary, the diagnosis of RPA-LA shunt is difficult and challenging. The unusual presentation of cyanotic congenital heart disease in late adolescent should also be considered a rare entity like RPA-LA shunt. Echocardiography, contrast-enhanced CT and selective angiographic studies are essential investigations for diagnosis and surgical closure of the shunt is the best available treatment modality.

## Consent

Written informed consent was obtained from the patient for publication of this Case report and any accompanying images.

## Abbreviations

BMI: body mass index
CT: computed tomography
PaO_2_: partial pressure of oxygen
RPA-LA: right pulmonary artery to the left atrium
